# European Consumers Attitudes toward Ethnic Foods: Case of Date Fruits

**DOI:** 10.3390/foods11152192

**Published:** 2022-07-23

**Authors:** Fatima El Hadad-Gauthier, Bleoussi Bernardin Monhoussou, Abdelhakim Hammoudi, Maria Angela Perito

**Affiliations:** 1 Institut Agronomique Méditerranéen de Montpellier (CIHEAM-IAMM), 3191 route de Mende CS 43999, CEDEX 5, 34093 Montpellier, France; elhadad@iamm.fr (F.E.H.-G.); bmmonbeb@gmail.com (B.B.M.); 2 National Research Institute for Agriculture, Food and the Environment (INRAE), AgroParisTech, Paris-Saclay Applied Economics, University of Paris-Saclay, 91120 Palaiseau, France; abdelhakim.hammoudi@inrae.fr; 3 Faculty of Bioscience and Technology for Food, Agriculture and Environment, University of Teramo, 64100 Teramo, Italy

**Keywords:** ethnic food, organic foods, consumers’ attitude, willingness to pay, date fruits

## Abstract

This study focuses on the perception of ethnic foods by European consumers. The aim of this work is to enrich the literature on the analysis of consumer perception of ethnic foods by focusing on the socio-demographic characteristics of consumers, the possible role played by product attributes, psychographic characteristics, and willingness to pay for these products, specifically date fruits. We surveyed a representative sample of 1123 Italian and French consumers. Using an ordered logit model, we found that, as for any other product, geographical indication, region of production, organic character, and fair trade are attributes that individuals consider in their purchase decisions for ethnic foods. Similarly, country of origin is a source of quality for ethnic foods such as dates. The results reveal that women, more educated individuals, and Generation Z (younger individuals) have a higher willingness to pay for organic, fair trade, and GI-labeled ethnic foods. Finally, this willingness to pay is driven more by product knowledge than by cognitive closeness to the ethnic food.

## 1. Introduction

Industrialization, urbanization, economic development, and globalization of markets have led to rapid changes in diets and lifestyles [1]. Food preferences across societies have evolved and offered differences between traditional and modern diets [2]. In this context, European consumer preferences have changed significantly in the last decades in terms of taste and belief [3]. There is a paradox between the globalization of tastes where individuals want to consume food from all over the world [4] and food sovereignty, where individuals prefer to eat food produced close to home [5,6]. One of the reasons consumers may want to buy imported products is that they have one or more unique attributes that entice to buy them instead of others. Moreover, their perception of better quality as opposed to domestic products has its explanation [4].

The literature shows that Europeans prefer domestic products to imported products in general. Nevertheless, there are some studies that show that for imported products, when looking at specific characteristics such as organic, fair-trade, and geographical indication, there is a high attention that is paid by European consumers [7,8]. However, very few studies have focused on European consumers’ perception of imported food products. Thus, we wonder what the different effects of labels (Organic, Geographical Indication, Fair Trade) on these foods and the preferences of European consumers towards these foods according to socio-demographic conditions and psychographic characteristics are.

There are studies that give indications on consumer preferences towards the imported food. Indeed, Thorgersen et al. [8] show on country samples (Germany, France, Denmark, China, and Thailand) a general preference for organics over conventional products. Alphonce et al. [7], considering dried fruits from Africa (bananas, pineapples, and mangoes), show that Norwegian consumers are willing to pay a premium for organic and fair-trade products. However, there are no studies to the best of our knowledge that integrate all three characteristics (Organic, Geographical Indication, and Fair Trade) in the analysis and use ethnic foods as we define them. This research does so by using an ethnic food: the date fruits.

Strictly speaking, ethnic foods are defined as originating from the heritage, the culture of an ethnic group, a group that uses their knowledge, local ingredients from plant and/or animal sources [9]. We consider ethnic products to be those grown with ancestral knowledge not produced in the country of consumption and which cannot be produced because of the specific characteristics of the food.

Few studies have investigated consumer behavior towards ethnic products and the effect of different labels on the preference of these products in Europe. Migliore et al. [10] investigate the factors affecting avocado consumption in Italy. The results of this study show that avocado consumption is affected by various factors, including fruit consumption habit, neophilia attitudes, and various intrinsic and extrinsic quality attributes (credibility attributes in particular).

The classical framework of consumer behavior proposes that food choices are the result of considering intrinsic (e.g., color, texture, and taste) [11,12] and extrinsic (e.g., brand, origin, and packaging) [13,14] factors, moderated by the demographic and socioeconomic characteristics of the consumer [7]. On this same theme, considering milk and pork, Thorgersen et al. [8] show a general preference for domestic over imported products and among imported foods a preference of foods from economically developed countries over less developed countries. This suggests that food’s country of origin matters. To go further, Sabbe et al. [15] show on a sample of Belgian consumers, a general positive attitude towards the consumption of tropical fruits such as avocado, coconut, dragon fruit, litchi, pineapple, mango, and papaya.

Studies in non-European countries also show variation in consumer preferences by country of origin for imported products. Menapace et al. [16] show that Canadian consumers’ willingness to pay for olive oil varies according to its origin country and that this willingness to pay is greater for products with a geographical indication label than for products without a geographical indication. Similarly, Ortega et al. [17], in a study of U.S. pork consumption in China, reveal that individuals’ age, location of purchase, and food safety concerns significantly influence their willingness to pay for U.S. pork. These studies show that consumers’ expectations and perceptions of food products from other countries can influence their willingness to pay for these products. Considering these different results, our point of view is that in the case of ethnic foods, there are differences in the willingness to pay for these ethnic foods according to the labels considered and the origin of the product. We also assume that there are differences in consumer perceptions of ethnic food quality between countries of origin according to socio-demographic groups.

Consumer preferences and behavior patterns are influenced by a variety of factors. In most studies of consumer attitudes and purchasing behaviors, the influence of demographic characteristics has been used to better explain the results and to determine the influence of personal characteristics on attitudes and behaviors, although this is not necessarily the primary objective of these studies [18]. While some of the studies reviewed showed no relationship between demographic characteristics and attitudes [19,20], other studies found significant effects of demographic variables. Indeed, Migliore et al. [10] show in a sample of Italian consumers that gender, education, and income influence the likelihood of consuming avocado more frequently. Thus, more educated Italian consumers and those with higher income levels tend to consume avocado more frequently. Moreover, women tend to be more familiar with tropical fruits and, therefore, consume avocados more frequently. In a sample of Norwegian consumers, Alphonce et al. [7] (2015) find that willingness to pay for African dried fruit is influenced by gender and education. Age is a very important variable in socio-demographic characteristics. However, some studies use generational cohorts instead of age since “cohort effects are lifelong effects” as identified by Schewe and Meredith [21]. On this basis, Kamenidou et al. [22] show that there are differences in the purchasing behavior of Greek consumers across generations. For example, baby boomers and Generation X purchase organic food more frequently than Generation Z and millennials. On this same basis, Perito et al. [23] show on a sample of Italian consumers of olives leaves that the drivers of willingness to accept food with upcycled ingredients were not monotonic with respect to the respondent’s age and each generation had distinctive characteristics that were not necessarily similar to the next generation. Terano et al. [24] in a study on local and imported fruit preferences in Malaysia show that household size, country of origin, and fruit variety are the variables that influence fruit preference among the younger generation.

In addition to socio-demographic characteristics, psychographic characteristics are variables that also influence consumer purchasing behavior. Several studies show the effect of psychographic characteristics on consumers’ purchasing behavior toward imported foods [25,26,27]. We wonder then, if there are differences in the perception of ethnic foods according to socio-demographic groups and psychographic characteristics.

The purpose of this work is to enrich this new stream of literature on the analysis of consumer perception of ethnic products by focusing on the socio-demographic characteristics of consumers by explicitly considering the possible role played by product attributes, psychographic characteristics, and willingness to pay for these products. For this, we investigate whether (a) consumers’ perceptions of ethnic product attributes differ according to socio-demographic and psychographic characteristics, (b) socio-demographic conditions and psychographic characteristics influence the willingness to pay for these ethnic products, (c) there are differences in consumers’ perceptions of the quality of ethnic products between different countries of origin according to socio-demographic groups, and (d) the willingness to pay for ethnic foods differs according to the country of origin and the labels considered.

## 2. Materials and Methods

To investigate how consumer choices of so-called ethnic products are determined in Europe, an online survey was conducted in France and Italy. The content validity of the questionnaire was ascertained using, first, a pre-test to collect elements to assess completeness and clarity of the questionnaire. In particular, we convened a limited audience (about 30 people) to understand if the survey presented potential inconsistencies. This allowed us to remove ambiguity in the questions. Second, a pilot survey on a sample of 50 people in order to verify the coordination between the survey and the correct functioning of the questionnaire. The responses obtained from the pilot survey were excluded from the overall data analyzed.

The final dissemination of the questionnaire was carried out by a survey agency (IPSOS) that guaranteed the representativeness of the samples in France and Italy. The final data of the French sample were collected during the month of July 2021. The data of the Italian sample were collected during the month of October 2021.

It is important to underline that the study did not require ethics committee approval for the survey. In particular, in the study, there was no actual or potential harm to the participants. Participants answered an anonymous online questionnaire. All personal data were treated anonymously, without any possibility on the part of the authors to identify the respondents. There was no way to identify the respondents from the data. The interviewed were informed that their participation was strictly on an anonymous and voluntary basis and explicitly gave their consent by clicking a specific button and accepting to continue with the interview. A detailed privacy statement was given, and the respondents could access the online questionnaire only after accepting the stated privacy policy. The study does not require any ex ante categorization of the participants by race/ethnicity, age, disease/disabilities, religion, sex/gender, sexual orientation, or other socially constructed groupings. No question on sensitive issues (e.g., health status, ethnicity, or religious belief) was asked.

The two countries, France and Italy, were chosen because of their specific characteristics. Indeed, they are two European date fruits importing countries, but their cultural proximity with Mediterranean countries differs. French and Italian consumers have different levels of knowledge about ethnic products. For Italian consumers, date fruits are considered a more ethnic product than for French consumers. Italy has lower cultural proximity to the Mediterranean countries than France. This is due to the presence in France of many second-generation migrant nationals from date-fruits producing countries.

The survey data were collected by interviewing 1000 individuals in France and 1000 individuals in Italy. We explicitly focus on the evaluations of current consumers and exclude non-buyers of the product under study. We therefore excluded 877 individuals and then had a total sample of 1123 European consumers. It is important to note that by excluding individuals who are not buyers, we are not able to assess whether they would derive any utility from the product. In that way, we are only exploring current perceptions of attributes by current consumers and not potential consumers or potential changes in perceptions that might be achieved through new information dissemination efforts.

The edited questionnaire aimed to investigate the perception of European consumers towards an imported food product, dates fruits specifically. The starting hypotheses were:

**H1.**  Perceptions of ethnic product attributes differ across socio-demographic groups.

**H2.**  Consumers’ socio-demographic conditions and psychographic characteristics influence their willingness to pay for date fruits.

**H3.**  Differences in consumer perceptions of ethnic food quality should exist between different countries of origin according to socio-demographic groups.

**H4.**  The willingness to pay for ethnic food differs according to the labels considered and the origin of the product.

The questionnaire used was therefore designed to test the hypotheses and is divided into three main parts. The first part focuses on the socio-demographic characteristics of the respondents (gender, age, work status, household size, education, and employment).

The second part focuses on the perception of the ethnic product (date fruits) and psychographic approach. In particular, this part of the questionnaire includes questions such as: “how often do you buy dates?” “How would you judge your knowledge of dates?” What is important to you when you buy date fruits: brand, variety, packaging, indication of origin, organic, fair trade, nutritional characteristics, or price?” The third part dealt with analyzing consumers’ willingness to pay for ethnic products. Specifically, this part of the questionnaire includes questions such as: “When you buy 250 g, what price are you willing to pay for these characteristics: brand, variety, packaging, indication of origin, organic, fair trade, nutritional characteristics, and price?” “When you buy 250 g of date fruits, what price are you willing to pay if they originate from Algeria, Tunisia, Iran, Morocco, Israel, Pakistan, Middle East (Saudi Arabia), or California (USA)?”

More in detail, Table 1 shows the statistics of the socio-demographic characteristics. According to Perito et al. [28], nine attributes were selected to assess the perceived importance in the decision to buy date fruits—brand, variety, packaging, geographical indication, organic, fair trade, nutritional characteristics, price, and region of production. The subjects of our survey were asked about their habits and quantity of date fruits purchased. Table 2 shows the statistics of the date fruits attributes selected.

Particular attention was paid to psychographic characteristics such as respondents’ knowledge of the product (self-awareness) and their involvement in date fruits consumption (Cognitive closeness) [28]. More specifically, subjects were asked to answer questions describing their personal knowledge of date fruits through a Likert-type scale from 1 to 5: “I have good knowledge about date fruits”; “I know how to judge the quality of date fruits”; “I can judge whether date fruits deserve their price or not”; “I have good knowledge of the nutritional characteristics of date fruits and their health benefits”. For involvement in date fruits consumption (Cognitive closeness about date fruits), respondents were asked to answer two questions assessed using a Likert 1 to 5 scale: “Date fruits have an important meaning for me”; “For me date fruits are a real passion”. Table 3 shows the statistics of the psychographic characteristics.

To identify whether there is a classification of date fruits quality according to the country of origin of the date fruits, respondents were asked to rank the following countries according to their expectations in terms of date fruits quality: Algeria, Morocco, Iran, Israel Pakistan, Tunisia, Middle East (Saudi Arabia), and California (USA).

To assess consumers’ willingness to pay for date fruits, participants were asked the price they would be willing to pay for 250 g of date fruits according to the type (branched date fruits, unbranched date fruits, and fresh date fruits), the label (date fruits with a geographical indication, organic date fruits, and date fruits with the fair-trade label) and the origin of the dates (Algeria, Morocco, Iran, Israel, Pakistan, Tunisia, Middle East, and California). For the choice of the price range, we checked the prices on the shelves of the main food retailers in Italy and France, on the wholesale markets, and on the internet portals (e.g., Amazon, Cora, Leclerc, etc.)

Consumer choices of date fruits are modelled using a discrete choice model based on random utility, the ordered logit [29,30,31].

Unlike the OLS which treats the variable as if it were continuous or the multinomial logit which treats the variable as a nominal variable, we chose the ordered logit since it allows the ordinality of the attributes to be considered. As mentioned above, the attributes were evaluated using a Likert- scale from 1 to 5 which measures the degree of importance of each attribute. The ordered logit model assumes the existence of a latent variable—an unobservable and unmeasurable variable. This latent variable, in our case consumer preference, is expressed in terms of observable explanatory variables such as socio-demographic characteristics.

The estimated model is therefore in the form:

(1) $$Y_{i}^{*}=\overset{K}{\underset{k=1}{\sum }}\beta_{k}X_{ki}+\varepsilon_{i}$$

where $Y_{i}^{*}$ represents the latent variable associated with the importance of the attributes, $\beta_{k}$ the estimated parameters, $X_{ki}$ the explanatory variables of the model, and $\varepsilon_{i}$ the error term. This model is estimated by the maximum likelihood method.

The Probit variant of this model was also tried, but its predictive power was inferior. According to Desaigues and Point [32], two properties make the logistic function interesting for modelling discrete choices: its reduced interval to 0–1; the logit function can be used as a probabilistic function, and it has the possibility of being linearized by log transformation.

## 3. Results

In the following we first present the results of an overall analysis of consumer choice based on date fruits attributes. We then focused our analysis on the label attributes (fair trade, organic, and GI) using the willingness to pay, and finally, we examine the effect of country of origin on the perception of ethnic products.

We recall that the sample size is 1123 individuals after excluding all non-buyers of date fruits. This means that we have excluded 43% of the base sample, and therefore, there are not so many people who buy dates. Moreover, the first analysis shows that there is no statistical difference between the two countries considered in the study.

Table 4 presents the estimates of the ordered logit model for the nine date attributes considered. In an ordered logit model, the signs of the estimated coefficients have to be interpreted as the variation direction of the dependent variable according to the increase in the regressor. In other words, the estimated positive coefficient indicates that as the regressor increases, the probability of being in the lowest category (low) decreases, while the probability of being in the highest value category (high) increases.

The analysis shows that all the attributes considered are significant. Cronbach’s alpha tests were performed on these attributes to assesses their reliability. It can be seen that the effect of socio-demographic groups on the attributes differs from one attribute to another. It is noted that the gender variable positively affects the nutritional characteristic criterion while the household size affects the Brand and Fair-Trade attributes. This means that women give greater importance to the nutritional characteristic of the date fruits than men, and the larger the household, the more likely it is to give greater importance to the brand of dates and the fact that the date is fair trade. It is also remarked that older respondents, i.e., those from the baby boomer generation and those with higher levels of education, place less importance on brand, variety, and price attributes than their younger and less educated counterparts.

In relation to the respondents’ employment, there is a difference in the perception of the attributes according to each modality of the variable. For example, employed respondents gave high importance to the attributes organic, geographical indication, fair trade, nutritional characteristic, and brand, whereas retired respondents were only interested in geographical indication.

Overall, these results show that there are differences in the way demographic groups perceive ethnic products.

Table 5 shows the ordered logistic regression for the willingness to pay according to the labels. In this section, we regress the willingness to pay for labelled ethnic food on socio-demographic and psychographic characteristics.

We notice that gender positively affects organic dates and fair-trade dates. This means that women have a higher willingness to pay for organic and fair-trade ethnic food than men.

Millennials are the only generation that affects willingness to pay by label. We observe that the sign is negative for each label. This implies that the willingness to pay for labelled ethnic food of millennials is lower than that of generation Z.

In relation to the employment of the respondents, we perceive a positive effect of individuals who work on the willingness to pay for dates of all labels and a positive effect of retirees only on dates labelled GI. Thus, employed individuals have a high willingness to pay for ethnic food of all types of labels, while retirees are only interested in ethnic food with a GI label.

In addition to socio-demographic characteristics, psychographic characteristics (knowledge and cognitive closeness) also affect willingness to pay. Indeed, individuals with a high knowledge of dates showed a higher willingness to pay for all types of labels, but those with a cognitive closeness to dates had no major effect on willingness to pay.

Consumers with cognitive closeness to dates showed a higher willingness to pay for dates from all origins, but those with high knowledge of dates had a higher willingness to pay only for dates from specific countries including Morocco, Israel, and the Middle East (Table A1).

There is a variation in the willingness to pay according to the different labels considered. Indeed, compared to individuals with a low level of education, individuals with a high level of education have an availability to pay whose probability increases whatever the type of label. This probability increases by 38.1%, 37.8%, and 41.8%, respectively, for GI, organic, and fair-trade dates.

Similarly, individuals with a job have a higher probability of being willing to pay, which differs according to the type of label. These probabilities are 67.4%, 52.2%, and 42.9%, respectively, for GI, organic, and fair-trade dates.

The willingness to pay for GI dates is higher than for organic dates for the most educated and employed individuals.

In sum, the willingness to pay for dates differs according to the labels considered.

The analysis according to the country of origin of the dates shows that gender and education level do not bring a significant difference in the perception that individuals have of the quality of dates according to their origin (Table A2).

Household size increases the probability that individuals have a better perception of date quality for dates from Algeria and the Middle East but decreases this probability for dates from Israel.

However, compared to unemployed individuals, employed individuals and retirees have a significant and positive effect on the perception of the quality of dates from Israel. They therefore judge that date fruits from Israel are of better quality.

Moreover, there is a difference in the willingness to pay according to the origin of the dates. For example, the probability of having a higher willingness to pay for students increases by 78.5% for dates from Pakistan and 55.9% for dates from California.

Thus, it is noted that there are differences in consumers’ perceptions of the quality of dates from various countries of origin according to socio-demographic characteristics, and the willingness to pay for dates differs according to the origin of the dates.

## 4. Discussion

The results showed that all the stated hypotheses were validated and that ethnic food consumption, as date fruits, is affected by different factors. Many of them relate to the consumer’s perception of certain quality attributes. A major effect is shown by three credibility attributes, geographical indication, fair trade, and organic certification, highlighting that consumers assign a high level of importance to these attributes when deciding to consume ethnic foods.

This result confirms the ones of other studies, showing that geographical indication is important in inferring food quality. Knowledge of the products’ origin is a factor that can potentially alter consumer perception of these products [33]. The literature indicates that consumers consider the origin of a food to be synonymous with high quality, although this may change depending on the product under consideration and the geographical context [13,14,34,35].

The literature also informs us that fair trade is an attribute used by consumers in purchasing decisions. Studies of consumer preferences for fair-trade products have found empirical evidence of willingness to pay for these products [36,37,38]. Indeed, consumers who purchase the most expensive fair-trade products reveal their preference for ethical characteristics and, as a result, they derive additional utility from them [39].

According to the literature, organic certification is also considered as a significant predictor of consumer choice. The results of many empirical studies show a positive causal relationship between production processes and perceived environmental effects. This is the case for organic food products, which consumers associate with natural processes and the non-use of pesticides and fertilizers [40], all of which have a positive effect on environmental sustainability and health [41,42].

This study highlights a higher willingness to pay for ethnic foods with these three attributes. This result is in line with the work of Menapace et al. [16] who underline that Canadian consumers’ willingness to pay for olive oil is higher for products with a geographical indication label than for products without a geographical indication label and the work of Tagbata and Sirieix [43] who using chocolate show that organic and fair-trade labels increased French consumers’ willingness to pay. Loureiro and Lotade [44] also point those consumers were willing to pay more for fair-trade coffee.

This study also confirms that, as with any other food, there are differences in consumer perception of date fruits from different countries. This result is in line with the work of Thorgersen et al. [8] who show a varied preference for milk and pork by consumers depending on the countries considered in their samples, as well as the work of Gao et al. [45], showing that French consumers preferred fresh fruit from Spain over that from China. Indeed, consumers differentiate between countries of origin, as they believe that the country of origin of the food is related to quality, along with the brand, price, and different product labels [46,47].

Regarding socio-demographic characteristics, this study indicates that there are differences in the way demographic groups perceive date fruits. This contrasts to some extent with the work of Zepeda and Li [19] (2006) and Åsebø et al. [20], who find no relationship between demographic characteristics and consumer attitudes. Indeed, we note that women have a higher willingness to pay for organic and fair-trade ethnic foods than men. This result is consistent with several studies in the literature saying that women are more altruistic and responsive to sustainable products [7,48,49,50]. Indeed, Blanc et al. [51] showed that young students’ behavior in choosing honey is gender sensitive and that women are the group most attracted to sustainable products. This sensitivity towards the environmental sustainability of the product may be due to women’s involvement in sustainable production and food-related activities [52,53]. Additionally, as Smith and Brower [54] indicate, young women are more interested in purchasing “green” products than young men. Moreover, women’s attitude towards sustainable products could be interpreted as an altruistic sentiment [55]. However, this conjecture is countered by Wandel and Bugge [56], who found that men were willing to pay a higher premium for organic products than women, despite women’s greater interest in environmental or green products.

The generational results reveal that millennials have a lower willingness to pay for labeled ethnic foods than Generation Z (the youngest). This result is corroborated by Blanc et al. [50] who find that younger consumers (Generation Z) are more attracted to organic and environmentally sustainable products. However, by analyzing the frequency of purchase of organic products, Kamenidou et al. [22] show that millennials (Generation Y) occasionally purchase organic food and that older generations (Generation X and Baby Boomers) are the most engaged in purchasing organic food.

Results in relation to education level underline that more educated individuals have a greater willingness to pay for labelled date fruits. This result is in line with Alphonce et al. [7], who find that more educated individuals are willing to pay more for credibility attributes, especially the organic one. This is likely due to the fact that consumers with higher levels of education are more health conscious, show more concern for the environment, and at the same time enjoy higher purchasing power [57]. These results are consistent with the literature on willingness to pay for organic products, where educated consumers seem to care more about organic products than less educated ones [58].

In addition to socio-demographic characteristics, psychographic characteristics were found to be factors affecting willingness to pay for ethnic foods. This finding is consistent with several studies in the literature that show psychographic variables play a key role in consumer attitudes and perceptions [26,59,60,61]. Our results show that willingness to pay for labelled ethnic foods is driven more by product knowledge than by cognitive closeness to the product. This is in line with the work of Ferrari et al. [62] who find in a sample of young Belgian and Dutch consumers that well-informed consumers were more willing to accept genetically modified foods.

## 5. Conclusions

The European Union is the largest importer of ethnic foods such as date fruits in the world. As results, it is important to understand the European consumers’ preferences for this type of foods from different countries. Therefore, this study examined the attitude of European consumers towards imported products and specifically ethnic foods.

This study contributed to the theoretical understanding of consumer’s responses to multiple quality cues and more specifically of consumer preferences for fair-trade, organics, geographical indication, and country-of-origin effects. It shows that consumers assign a high level of importance and are willing to pay more for ethnic foods with these labels.

The fact that consumers are willing to pay more for food from one origin than another confirms that country-of-origin is indeed used as a quality cue. For food exporters, an important implication of this study is that country-of-origin image and various certification labels matter in consumers’ food choices, even when the product concerned is an ethnic food that is not a cultural product of the country of consumption.

Moreover, beyond understanding the behavior of French and Italian consumers with respect to ethnic foods, our empirical results may prove useful in refining the strategies of actors in developing countries in terms of promoting ethnic products in international markets. Indeed, on the one hand, our results give an idea of the consumer profiles’ sensitivity to the different signs of quality and those whose targeted marketing campaigns may be necessary to conquer new markets. On the other hand, our results can help guide public policies to support development and marketing to respond to new market opportunities for ethnic foods.

Additionally, our empirical results for French and Italian consumers motivate several important avenues of research to better understand consumption behavior toward ethnic foods. In fact, other methods such as choice experiments and non-hypothetical choice experiments could be used for these studies to better understand consumer behavior.

However, the study shows some limitations that could be considered in future research. In fact, in the survey, only verbal descriptors have been used to identify the European consumer perception and willingness to pay for ethical food products, which might mimic a real market in a less realistic way. Moreover, to test the hypotheses regarding the acceptance of ethnic products, only two markets (Italy and France) and one product (date fruits) were tested. From our point of view, further research should (i) simulate real shopping environments, where the choice sets are designed with real image and sensorial analysis to increase the accuracy of the results, and (ii) consider more markets and products.

## Figures and Tables

**Table 1:** Socio-demographic characteristics of the sample, *n* = 1123.

Variable	Variable Definition	Count	% of Sample
**Gender**	Male	512	45.59
	Female	611	54.41
**Generation**	Generation Z	120	10.69
	Millennials	138	12.29
	Generation X	445	39.63
	Baby-boomers	420	37.40
**Education**	Low	285	25.38
	Middle	425	37.85
	High	413	36.78
**Employment**	Unemployed	117	10.42
	Student	68	6.06
	Homemaker	87	7.75
	Retired	216	19.23
	Worker	635	56.54
**Household size**	One person	152	13.54
	Two persons	359	31.97
	Three persons	294	26.18
	Four persons	223	19.86
	Five persons	72	6.41
	More than five persons	23	2.05

**Table 2:** Date fruits attributes importance (scale: 1—Not important; 2—Somewhat important; 3—Slightly important; 4—Important; and 5—Very important).

Variable	Mean	Standard Deviation
*n =* 1123		
Brand	3.15	1.05
Variety	3.57	0.97
Packaging	3.43	0.99
Geographical indication (IG)	3.79	0.97
Organic	3.47	1.12
Fair trade	3.49	1.05
Nutritional characteristic	3.69	1.00
Price	3.89	0.86
Region of production	3.80	0.92

**Table 3:** Psychographic variables.

Variable	Modalities	Mean	Standard Deviation
Product knowledge	I have a good knowledge about date fruits	3.04	1.06
	I know how to judge the quality of date fruits	3.17	1.06
	I can judge whether date fruits deserve their price or not	3.22	1.05
	I have a good knowledge of the nutritional characteristics of date fruits and their health benefits	3.34	1.06
Cognitive closeness	Date fruits have an important meaning for me	2.88	1.16
	For me date fruits are a real passion	2.95	1.17

**Table 4:** Ordered logit model estimates for the nine date attributes considered.

	(1)	(2)	(3)	(4)	(5)	(6)	(7)	(8)	(9)
Attributes	Brand	Variety	Packaging	IG	Organic	Fair Trade	Nutritional Characteristic	Price	Region of Production
.									
Household size	0.138 ***	0.0427	0.0189	−0.0276	0.0653	0.116 **	0.0470	0.0433	0.0144
	(0.0483)	(0.0480)	(0.0486)	(0.0483)	(0.0477)	(0.0484)	(0.0488)	(0.0499)	(0.0482)
1. Generation Z									
2. Millenials	−0.249	−0.395	0.0200	−0.353	0.0283	−0.303	−0.0818	0.292	−0.144
	(0.275)	(0.274)	(0.282)	(0.281)	(0.270)	(0.275)	(0.277)	(0.290)	(0.274)
3. Generation X	−0.543 **	−0.598 **	−0.143	−0.0702	−0.193	−0.185	−0.208	0.267	−0.0639
	(0.254)	(0.252)	(0.257)	(0.257)	(0.249)	(0.253)	(0.257)	(0.265)	(0.248)
4. Baby-boomers	−0.647 **	−0.722 ***	−0.255	0.124	−0.109	−0.220	−0.0183	0.123	−0.0848
	(0.273)	(0.272)	(0.276)	(0.276)	(0.268)	(0.271)	(0.275)	(0.284)	(0.268)
Gender (female)	−0.129	−0.0757	0.129	0.158	−0.0517	0.0436	0.269 **	−0.0196	0.0531
	(0.115)	(0.115)	(0.115)	(0.117)	(0.114)	(0.115)	(0.116)	(0.119)	(0.116)
1. Employment (Unemployed)									
2. Student	−0.466	−0.795 **	−0.469	0.453	−0.0296	0.290	−0.0569	−0.156	−0.646 *
	(0.346)	(0.347)	(0.363)	(0.355)	(0.345)	(0.347)	(0.350)	(0.362)	(0.354)
3. Homemaker	0.878 ***	0.950 ***	0.635 **	1.294 ***	0.961 ***	1.094 ***	0.641 **	−0.353	0.707 ***
	(0.273)	(0.281)	(0.273)	(0.279)	(0.271)	(0.274)	(0.278)	(0.282)	(0.274)
4. Retired	0.269	0.313	−0.126	0.503 **	0.166	0.202	−0.168	−0.765 ***	0.0894
	(0.232)	(0.236)	(0.235)	(0.235)	(0.232)	(0.231)	(0.237)	(0.242)	(0.236)
5. Worker	0.520 ***	0.359 *	0.0590	0.745 ***	0.724 ***	0.511 ***	0.390 **	−0.364 *	0.229
	(0.191)	(0.194)	(0.196)	(0.194)	(0.192)	(0.191)	(0.198)	(0.200)	(0.195)
1. Education (low)									
2. Middle	0.0400	0.0995	0.136	0.165	0.190	0.119	0.457 ***	−0.268 *	0.131
	(0.148)	(0.149)	(0.148)	(0.151)	(0.147)	(0.148)	(0.148)	(0.153)	(0.151)
3. High	−0.226	−0.0825	−0.101	−0.0350	0.0437	0.0252	0.115	−0.460 ***	0.191
	(0.152)	(0.152)	(0.151)	(0.154)	(0.150)	(0.152)	(0.151)	(0.156)	(0.154)
*N*	1123	1123	1123	1123	1123	1123	1123	1123	1123
pseudo *R*^2^	0.015	0.010	0.009	0.014	0.013	0.013	0.016	0.011	0.008

Standard errors in parentheses; * *p* < 0.10, ** *p* < 0.05, *** *p* < 0.01.

**Table 5:** Ordered logistic regression for the willingness to pay according to the labels.

Labels	(1)	(2)	(3)
	(IG)	(Organic)	(Fair Trade)
Household size	0.0311	0.140 ***	0.0261
	(0.0481)	(0.0479)	(0.0474)
1. Generation Z			
2. Milllenials	−0.603 **	−0.486 *	−0.444 *
	(0.264)	(0.261)	(0.268)
3. Generation X	−0.0813	−0.0951	−0.147
	(0.241)	(0.238)	(0.242)
4. Baby-boomers	−0.119	0.0159	−0.0107
	(0.261)	(0.256)	(0.259)
.			
Gender (female)	0.139	0.241 **	0.229 **
	(0.112)	(0.111)	(0.111)
1. Employment (Unemployed)			
2. Student	0.236	0.294	0.232
	(0.340)	(0.331)	(0.337)
3. Homemaker	0.0162	−0.0576	−0.140
	(0.274)	(0.267)	(0.266)
4. Retired	0.530 **	0.274	0.139
	(0.231)	(0.228)	(0.227)
5. Worker	0.674 ***	0.522 ***	0.429 **
	(0.190)	(0.185)	(0.186)
1. Education (low)			
2. Middle	−0.175	0.0370	0.0326
	(0.147)	(0.146)	(0.143)
3. High	0.381 **	0.378 **	0.418 ***
	(0.149)	(0.148)	(0.147)
Product knowledge	0.210 ***	0.299 ***	0.180 **
	(0.0774)	(0.0768)	(0.0773)
Cognitive closeness	0.0686	−0.000673	0.0680
	(0.0667)	(0.0663)	(0.0663)
*n*	1123	1123	1123
pseudo *R*^2^	0.018	0.017	0.012

Standard errors in parentheses; * *p* < 0.10, ** *p* < 0.05, *** *p* < 0.01.

## Data Availability

The data supporting the findings of this study are available from the corresponding author (M.A.P.) upon reasonable request.

## Appendix A

**Table A1:** Ordered logistic regression for the willingness to pay according to the origin of the date fruits.

	(1)	(2)	(3)	(4)	(5)	(6)	(7)	(8)
	Algeria	Morocco	Iran	Israel	Pakistan	Tunisia	Middle East	California
Household size	0.104 **	0.0793 *	0.0689	0.0786 *	−0.0375	0.0680	0.109 **	0.0410
	(0.0465)	(0.0478)	(0.0473)	(0.0468)	(0.0477)	(0.0463)	(0.0462)	(0.0472)
1. Generation (generation Z)								
2. Milllenials	−0.855 ***	−1.015 ***	−0.342	−0.500 *	−0.226	−0.664 **	−0.450 *	0.0454
	(0.270)	(0.276)	(0.258)	(0.269)	(0.260)	(0.265)	(0.263)	(0.268)
3. Generation X	−0.499 **	−0.552 **	−0.153	−0.134	0.221	−0.467 *	−0.344	0.153
	(0.249)	(0.256)	(0.237)	(0.251)	(0.239)	(0.242)	(0.241)	(0.249)
4. Baby-boomers	−0.545 **	−0.512 *	−0.256	−0.00114	0.247	−0.497 *	−0.493 *	0.0978
	(0.268)	(0.274)	(0.256)	(0.270)	(0.259)	(0.261)	(0.260)	(0.267)
Gender (female)	0.162	0.167	0.211 *	0.116	0.0703	0.195 *	0.147	0.161
	(0.112)	(0.112)	(0.112)	(0.112)	(0.111)	(0.112)	(0.112)	(0.112)
1. Employment (Unemployed)								
2. Student	0.252	−0.163	0.261	0.274	0.785 **	−0.0524	−0.110	0.559 *
	(0.342)	(0.346)	(0.328)	(0.342)	(0.334)	(0.336)	(0.333)	(0.338)
3. Homemaker	0.00285	−0.273	−0.0951	−0.0728	0.167	−0.0998	−0.122	0.378
	(0.272)	(0.266)	(0.264)	(0.270)	(0.267)	(0.265)	(0.259)	(0.261)
4. Retired	0.406 *	0.260	0.249	0.315	0.521 **	0.0305	0.144	0.243
	(0.233)	(0.230)	(0.230)	(0.231)	(0.230)	(0.230)	(0.228)	(0.230)
5. Worker	0.591 ***	0.547 ***	0.597 ***	0.584 ***	0.871 ***	0.382 **	0.468 **	0.834 ***
	(0.192)	(0.189)	(0.188)	(0.190)	(0.192)	(0.190)	(0.184)	(0.190)
1. Education (low)								
2. Middle	−0.0238	−0.0880	−0.0939	0.00942	−0.0759	−0.212	−0.146	−0.231
	(0.145)	(0.145)	(0.145)	(0.145)	(0.146)	(0.146)	(0.144)	(0.144)
3. High	0.341 **	0.257 *	0.261*	0.339 **	0.261 *	0.114	0.256 *	0.0389
	(0.148)	(0.147)	(0.148)	(0.148)	(0.150)	(0.148)	(0.148)	(0.148)
Product knowledge	0.0688	0.168 **	0.116	0.210 ***	0.112	0.0845	0.135 *	0.0677
	(0.0762)	(0.0766)	(0.0770)	(0.0772)	(0.0766)	(0.0766)	(0.0771)	(0.0778)
Cognitive closeness	0.148 **	0.0833	0.119 *	0.0880	0.100	0.125 *	0.186 ***	0.150 **
	(0.0662)	(0.0670)	(0.0667)	(0.0668)	(0.0660)	(0.0659)	(0.0663)	(0.0666)
*n*	1123	1123	1123	1123	1123	1123	1123	1123
pseudo *R*^2^	0.016	0.017	0.015	0.015	0.015	0.012	0.019	0.015

Standard errors in parentheses; * *p* < 0.10, ** *p* < 0.05, *** *p* < 0.01.

**Table A2:** Ordered logit model estimates for the for the date fruits quality according to the origin.

	(1)	(2)	(3)	(4)	(5)	(6)	(7)	(8)
	Algeria	Morocco	Iran	Israel	Pakistan	Tunisia	Middle East	California
Household size	0.150 ***	0.0373	−0.0110	−0.0543	0.0559	0.0397	0.176 ***	−0.0232
	(0.0477)	(0.0468)	(0.0470)	(0.0478)	(0.0470)	(0.0476)	(0.0480)	(0.0473)
1. Generation (generation Z)								
2. Millenials	0.00630	−0.265	−0.00262	−0.130	−0.506 *	−0.473 *	−0.253	0.0575
	(0.266)	(0.266)	(0.265)	(0.265)	(0.259)	(0.264)	(0.261)	(0.257)
3. Generation X	−0.115	−0.382	−0.173	−0.0520	−0.558 **	0.0587	−0.389	0.0113
	(0.243)	(0.241)	(0.242)	(0.241)	(0.236)	(0.239)	(0.237)	(0.234)
.								
4. Baby-boomers	−0.0782	−0.206	−0.0395	0.0531	−0.708 ***	0.106	−0.261	0.218
	(0.263)	(0.260)	(0.262)	(0.259)	(0.257)	(0.259)	(0.257)	(0.253)
Gender (female)	−0.128	−0.178	0.177	0.0212	0.000332	−0.129	−0.0581	−0.0247
	(0.115)	(0.113)	(0.115)	(0.114)	(0.114)	(0.114)	(0.114)	(0.113)
1. Employment (Unemployed)								
2. Student	−0.359	−0.407	−0.0548	−0.220	−0.349	0.124	0.0766	0.217
	(0.339)	(0.347)	(0.334)	(0.343)	(0.334)	(0.338)	(0.330)	(0.335)
3. Homemaker	0.124	0.175	0.213	0.614 **	0.0277	0.449 *	0.546 **	0.629 **
	(0.268)	(0.268)	(0.263)	(0.264)	(0.262)	(0.265)	(0.268)	(0.262)
4. Retired	−0.0848	−0.0204	0.160	0.398 *	−0.117	0.0341	0.0230	−0.262
	(0.239)	(0.236)	(0.236)	(0.236)	(0.236)	(0.234)	(0.236)	(0.231)
5. Worker	0.0686	0.0994	0.256	0.437 **	−0.182	0.313	0.200	0.412 **
	(0.196)	(0.192)	(0.192)	(0.192)	(0.191)	(0.192)	(0.191)	(0.187)
1. Education (low)								
2. Middle	−0.132	0.194	−0.0259	0.176	−0.137	−0.118	−0.0929	−0.0287
	(0.148)	(0.146)	(0.145)	(0.145)	(0.146)	(0.146)	(0.144)	(0.146)
3. High	−0.188	−0.145	−0.118	0.0197	−0.200	−0.408 ***	−0.126	−0.501 ***
	(0.153)	(0.152)	(0.151)	(0.151)	(0.152)	(0.151)	(0.151)	(0.152)
*n*	1123	1123	1123	1123	1123	1123	1123	1123
pseudo *R*^2^	0.006	0.005	0.002	0.006	0.005	0.010	0.009	0.012

Standard errors in parentheses; * *p* < 0.10, ** *p* < 0.05, *** *p* < 0.01
